# Mesostructure of Ordered Corneal Nano-nipple Arrays: The Role of 5–7 Coordination Defects

**DOI:** 10.1038/srep28342

**Published:** 2016-06-22

**Authors:** Ken C. Lee, Qi Yu, Uwe Erb

**Affiliations:** 1 Department of Materials Science and Engineering, University of Toronto, 184 College St. Toronto, Ontario, M5S3E4, Canada

## Abstract

Corneal nano-nipple structures consisting of hexagonally arranged protrusions with diameters around 200 nm have long been known for their antireflection capability and have served as biological blueprint for solar cell, optical lens and other surface designs. However, little is known about the global arrangement of these nipples on the ommatidial surface and their growth during the eye development. This study provides new insights based on the analysis of nano-nipple arrangements on the mesoscale across entire ommatidia, which has never been done before. The most important feature in the nipple structures are topological 5- and 7-fold coordination defects, which align to form dislocations and interconnected networks of grain boundaries that divide the ommatidia into crystalline domains in different orientations. Furthermore, the domain size distribution might be log-normal, and the domains demonstrate no preference in crystal orientation. Both observations suggest that the nipple growth process may be similar to the nucleation and growth mechanisms during the formation of other crystal structures. Our results are also consistent with the most recently proposed Turing-type reaction-diffusion process. In fact, we were able to produce the key structural characteristics of the nipple arrangements using Turing analysis from the nucleation to the final structure development.

The corneal surface structures of many insect eyes have been studied for several decades (e.g. refs 1, 2, 3, 4, 5, 6, 7, 8, 9, 10). One specific case of surface structure has drawn particular attention: the nano-nipple arrays observed on many butterfly and moth eyes. They consist of millions of nano-sized protrusions arranged on the surface of the ommatidia in the insect compound eyes. The nipples of chitinous material have various diameters and heights in the size range of tens to a few hundreds of nanometers^4^,^6^,^9^. They can be arranged randomly or in highly ordered 2D crystal patterns, often with hexagonal close-packed arrangement^4^,^8^.

From a biological point of view, the influence of nipple structures on insect eyes has been discussed in numerous studies, and it has been proposed that they can serve several purposes including i) better vision in low light conditions by reducing light reflection on corneal surfaces^2^,^4^,^6^, ii) improved camouflage^6^ as well as iii) reduction of water wettability by creating superhydrophobic eye surfaces^11^.

In terms of technological importance, highly ordered nipple arrangements have been used as a biological blueprint to produce antireflective structure on polymeric, glass, or semiconductor surfaces for applications such as optical lenses and solar cells (e.g. refs 12, 13, 14, 15). Many different processes including etching, templating, lithography, or direct replica casting have been developed to make engineered surfaces, and several products have already been commercialized. However, it should be pointed out that reduced light reflection is not only observed for regular nipple structures, but also in irregular arrangements^16^,^17^.

Surprisingly, very little is known about the structural evolution of nipples during metamorphosis in the insect eye development. Gemne^5^ used extensive electron microscopy analysis to follow the nipple development over an 8-day pupation period of the moth *Manduca sexta* and concluded that the nipples are an integral part of the lens surface. The nipple development is initiated about 5 days after pupation with the formation of initial patches on the epicorneal lamina on top of underlying microvilli. This is followed by the anlage formation, first as low cupoles, then high cupoles after 6.5 to 7 days. After 7.5 to 8 days the high cupoles are filled with corneal substance through the microvilli and finally consolidate to form the final nipple structure. The arrangement of the microvilli tips in the eye cone was suggested to be in space filling hexagonal packing, explaining the observed arrangement of nano-nipples in roughly close-packed hexagonal domains^5^. The nipple arrangements showed many defects and the author assumed that the deviation from perfect hexagonal structure was due to the convex shape of the cornea.

However, a recent ground-breaking study proposed a completely different mechanism of nipple formation. Blagodatski *et al*.^8^ investigated a large number of corneal nanostructures in many different insect species, and they observed structures ranging from dimpled nanopatterns, irregular mazes, disordered nipples to highly ordered hexagonal nipple arrays across several lineages in 23 insect orders. They found striking similarities between the corneal nanostructures and the patterns generated by the Turing model, which is capable of modeling a multitude of microscale patterns observed in the animal kingdom^18^,^19^,^20^,^21^. Blagodatski *et al*.^8^ hypothesized that instead of secretion by microvilli, the formation of corneal nanostructures follows a similar process as described in the Turing reaction-diffusion model. This was likely the first study of biological Turing patterning on the nanoscale. Interestingly, Blagodatski *et al*.^8^ refer to these structures as surface coatings, with the highly ordered hexagonal pattern just being one solution among a variety of possible structures and pattern transitions.

Although both theories have their merits, neither is conclusive. Blagodatski *et al*.^8^ proposed that growth from space filling microvilli is questionable as it is only applicable to ordered corneal nipple structures found on some moth and butterfly eyes. Regularly spaced microvilli are unlikely to generate the multitude of irregular structures observed on other insect eyes. On the other hand, although the Turing model approach appears to be promising as a pattern formation mechanism, important details such as identities of the diffusing morphogens involved in pattern formation and their transport mechanisms, as well as the role of the microvilli in this process remain unknown^8^.

Unfortunately most previous studies have concentrated their structural analysis on relatively small sections within an individual ommatidium (e.g. refs 5,6,8 and 9). It can be expected that much more insight will come from further analysis of the corneal nipple arrays in much greater detail than has been done in the past. From a structure point of view there are several key questions that need to be addressed for the case of ordered nano-nipple patterns. First, what are the crystal and defect structures in the nipple arrays on the nanoscale? Second, on the mesoscale, what are the domain/crystal sizes as well as the orientations between adjacent crystals/domains over an entire ommatidium? Third, can the structure information on both the nanoscale and mesoscale give us clues as to the nipple formation in terms of location and time?

For the example of the highly ordered hexagonal nipple structure observed on the Mourning Cloak butterfly (*Nymphalis antiopa*, a member of Lepidoptera), we have recently addressed the first question by looking at the nanostructure of perfect and defective nipple arrays^22^,^23^. While several defects (e.g. vacancies, fusion defects) were observed with low frequencies, one particular defect was found quite often in this structure: the 5–7 coordination defect. Individual 5 or 7 coordination defects were rare, and they have the character of disclinations. The combination of the two in the 5–7 configuration forms a dislocation, similar to dislocations in other 2D crystal structures with and without curvature (e.g. refs 24, 25, 26, 27, 28, 29, 30, 31, 32). The surprising result of the study was that the 5–7 coordination defects are aligned in rows forming grain boundaries between adjacent crystals/domains with perfect structure, also similar to grain boundaries in other 2D hexagonal structures (e.g. refs 33, 34, 35, 36, 37, 38, 39, 40, 41, 42, 43, 44, 45, 46, 47). In fact, understanding symmetry breaking concepts in nanostructure ordering is of significant interest recently. For example, it has been shown very recently that symmetry violation can lead to both domain structure formation in ordered nano-nipple arrays in a moth eye as well as local ordering in the amorphous nanodimple structure of a snake skin^48^.

The current study presents a unique insight into the nano-nipple structure of highly ordered arrays on the mesoscale by studying nipple arrangements over entire ommatidia. This has never been done before and provides a detailed analysis of domain size distribution and domain orientations within individual and between adjacent ommatidia.

## Results

**Figure 1:**
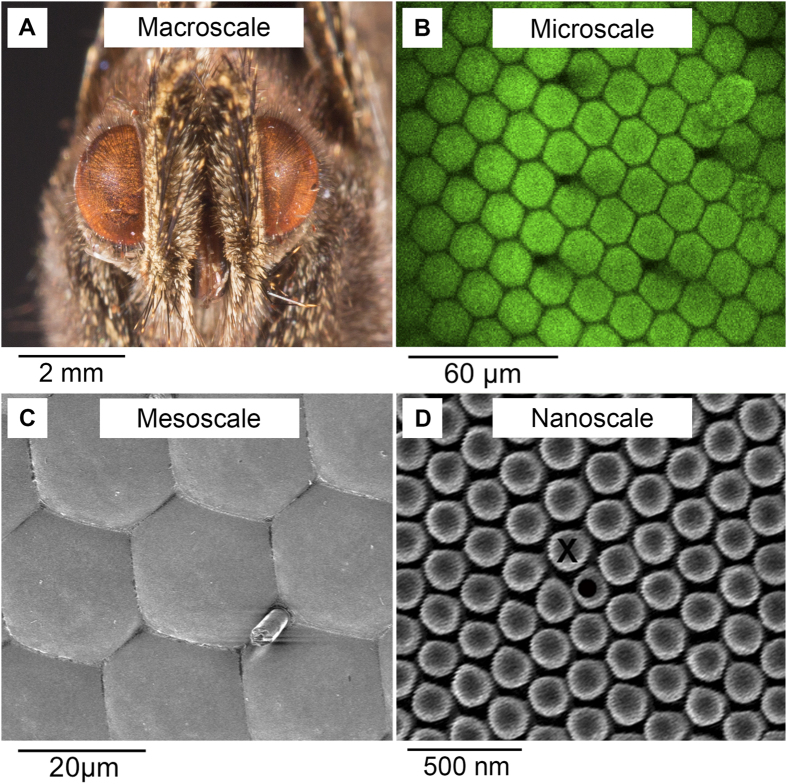
The eye of a Mourning Cloak butterfly on different length scales. (**A**) The head of the Mourning Cloak butterfly showing both semi-ellipsoidal eyes, (**B**) a confocal laser microscope image of the microscale ommatidial tessellation, (**C**) a mesoscale SEM image of ommatidia with a broken bristle at the junction between 3 facets, (**D**) the nano-nipple structure on the nano-scale showing one pair of 5–7 coordination defect labeled with “•” and “×” for 5- and 7-fold coordination defects, respectively.

To establish the structure level links on different length scales (macro, micro, meso, nano), Fig. 1, Fig. S1, as well as Table 1 summarize the major findings for the Mourning Cloak butterfly eye. The eye has roughly the shape of a semi-ellipsoid with major and minor axes of 2.2 mm and 1.6 mm, respectively (Fig. 1A). The total surface area is about 5 mm^2^ and the number of ommatidia is about 10400 (Table 1). Figure 1B is a confocal laser micrograph on the microscale clearly showing hexagonal tessellation with the individual ommatidia. Each facet has an area of about 500 μm^2^. The dark shadows in Fig. 1B are due to the protective bristles which are found occasionally at the junctions where three facets meet. The broken base of one bristle is more clearly seen in the SEM micrograph in Fig. 1C, showing the mesoscale structure of a few individual ommatidia. This structure level is the main focus of this study and will be analyzed in more detail below. Figure 1D shows a nanoscale micrograph with hexagonally arranged nipples and a single 5–7 coordination defect pair, marked with “•” and “×”.

In order to better understand the mesoscale analysis, Fig. S1A–D summarize the essential nanoscale characteristics of the nano-nipple structure, particularly the perfect hexagonal structure and the arrangement of 5–7 coordination defects to form a grain boundary between two crystals, in this case a low angle boundary.

**Figure 2:**
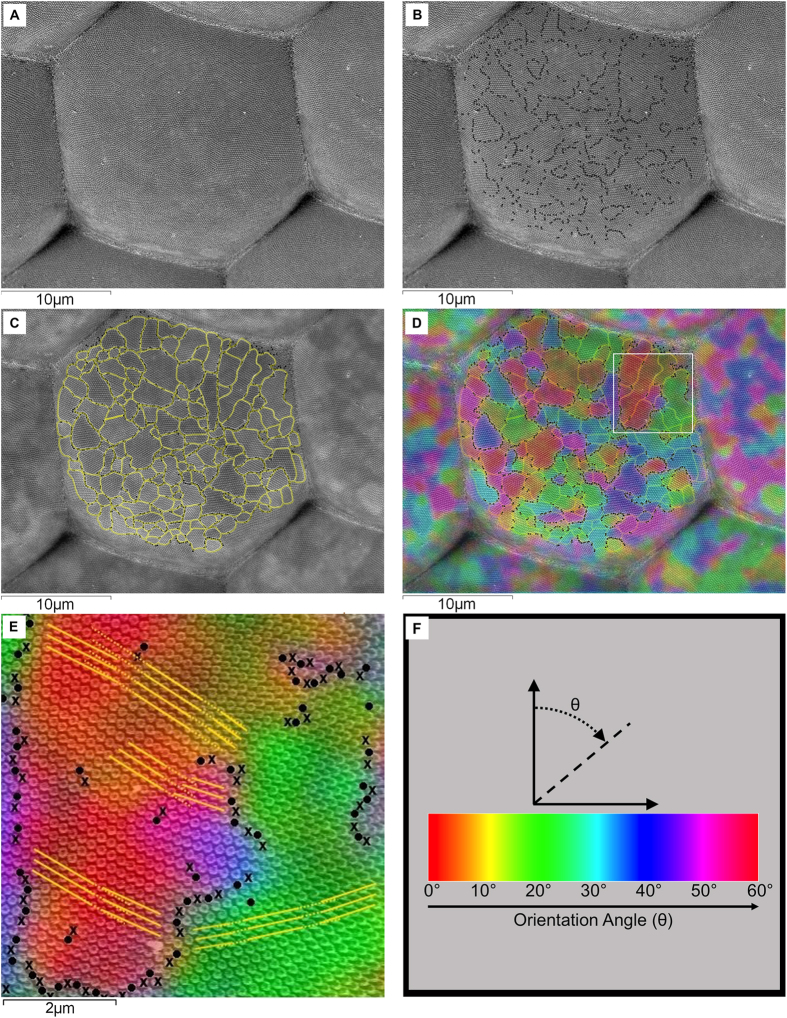
Ommatidium showing defined nipple crystals. (**A**–**D**) show the same ommatidium, and the nipple crystals become visible once the 5–7 defects are labeled (**B**). The crystal definition is further improved by tracing 5–7 defect rows with solid yellow lines (**C**). (**D**) shows the orientation map of the ommatidium, which is to be interpreted based on the color scale in (**F**). (**E**) is a close-up of the area outlined in white in (**D**), clearly showing the effectiveness of orientation mapping.

We now address the distribution of the 5–7 defects on the mesoscale by covering entire ommatidia. One particular example is shown in Fig. 2, and two other in Figs S2 and S3. Figure 2A shows a center ommatidium with six surrounding neighbors. Areas of perfect hexagonal nipple order with various sizes can be seen over the entire facet. The regions close to the facet boundaries between adjacent ommatidia show less than perfect order and were excluded from the following analysis. All 5–7 defect pairs distributed over the entire facet surface are marked in Fig. 2B. It can be seen that most of the 5–7 defect pairs are aligned in rows with varying distances between them forming an interconnected network of domain/crystal boundaries covering the entire facet. About 10% of all nipples are in defective 5–7 grain boundary positions. The network of grain boundaries effectively subdivides the facet into numerous grains/domains, each of which contains nipples in perfect hexagonal arrangement. In Fig. 2C the grain boundaries were highlighted by tracing the 5–7 defect rows with solid yellow lines. With the exception of a few slightly elongated grains, the grain structure is fairly equiaxed.

**Figure 3:**
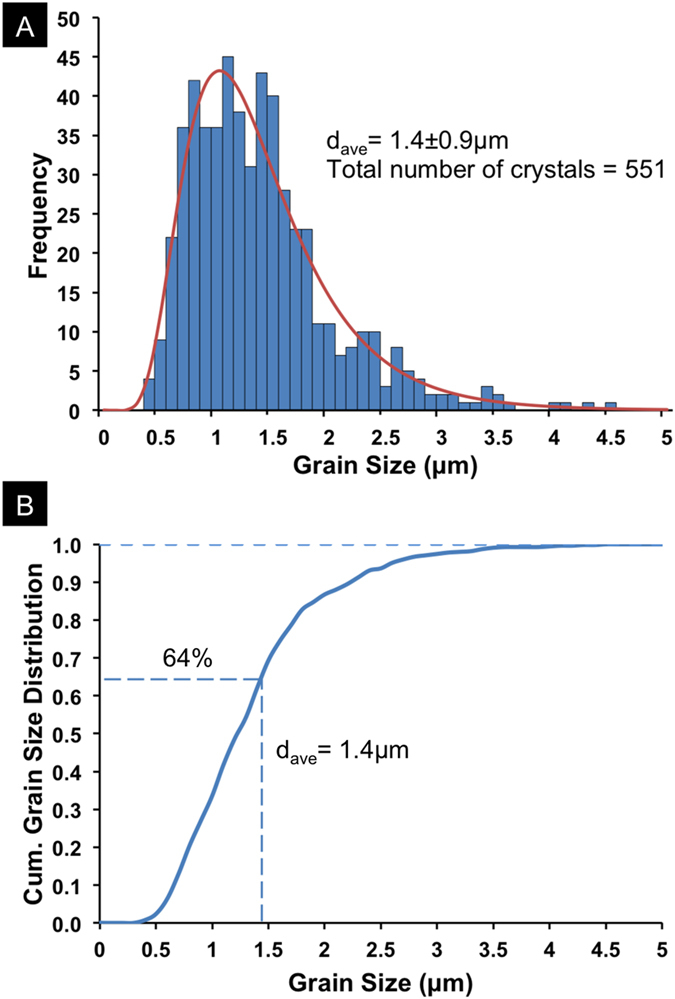
Nipple crystal size analysis showing (**A**) log-normal and (**B**) cumulative distributions of the nipple crystals with an average crystal size of 1.4 μm.

Nipple crystal size distribution was also determined based on Fig. 2C (See Materials and Methods), and the combined results for Figs 2, S2 and S3 are shown in Fig. 3. Figure 3A is the frequency distribution of crystal sizes showing an average crystal size of 1.4 μm. The bars represent the numbers of nipple crystals within different size range bins on the x-axis, with increments of 0.1 μm. The range containing the highest number of crystals is between 0.7 μm and 1.7 μm, and the numbers taper off for both larger and smaller crystal sizes. The largest grain size in the distribution was 4.6 μm. Notice the taper is more gradual for larger crystal sizes and sharper for smaller sizes; suggesting a log-normal distribution. The log-normal best-fit of the measured frequency distribution is given by the red curve in Fig. 3A.

Figure 3B is the cumulative size distribution for all nipple crystals measured. It shows the fraction of crystals that are smaller than or equal to a given size on the x-axis. For example, the dotted lines show that about 64% of all crystals in the distribution are smaller than the average grain size of 1.4 μm.

Figure 2D shows an orientation map where nipple crystals are color-coded by orientations using a plug-in^49^. It is interpreted based on the color scale (Fig. 2F), which shows different hues of colors for rows of closest packed nipples as a function of the orientation angle θ measured with respect to the top of the micrographs.

The figure clearly shows that color changes i.e. crystal misorientations are always associated with domain/grain boundaries consisting of rows of 5–7 defects with varying defect pair spacing, i.e. low angle and high angle grain boundaries. However, color/misorientation changes can also be observed in regions with lower 5–7 defect densities, e.g. the area outlined in Fig. 2D, shown at a higher magnification in Fig. 2E. In these cases the misorientation change is due to elastic bending of the crystal, in many instances due to the long distance strain field associated with nearby 5–7 pairs i.e. individual dislocations. Therefore the outlined boundaries in Fig. 2C are a mixture of domain/grain boundaries as well as misorientated regions due to lattice bending. Table 2 shows the total areas and percentages of ommatidium regions within the orientation ranges. The values range from about 14% up to 20%. In terms of crystallographic texture this means that there is no single preferred orientation which dominates over other orientations. In other words, the crystals in the ommatidium are arranged with a more or less random crystallographic texture. It should also be noted that there is no correlation between crystal orientations in adjacent ommatidia.

The results of the current study on the corneal nano-nipple structure of the Morning Cloak butterfly can be summarized as follows. First, the mesostruture of each ommatidium shows a multitude of grains/domains (on the order of about 120) of highly ordered hexagonal nipple arrangements. The grain/domain size distribution might be log normal. Second, the individual grains/domains are separated by grain boundaries which can be characterized as rows of 5–7 coordination defects in different configurations and varying spacings between the 5–7 pairs. Third, there is no strong preferred orientation in the crystals over an entire ommatidium. Fourth, at the facet boundaries and triple junctions between ommatidia there is very little order in the nipple arrangements. There are no indications of crystals or crystal orientations extending from one ommatidium to a neighboring ommatiduim.

The significance of the result is better understood in the context of crystallography. Both the log-normal crystal size distribution and the lack of preference in crystal orientation are characteristics of microstructures resulting from random nucleation and growth processes (e.g. refs 50, 51, 52). An example of such a process is the solidification of a simple liquid, where “nucleation” first takes place to form many small clusters of atoms in randomly oriented nuclei. As solidification proceeds, the nuclei become preferential points for crystal “growth,” which ultimately coalesce to complete the crystalline solid^53^,^54^ usually with no preferred orientations of the grains. However, the growth of the nipple crystals is likely much more complex than this.

**Figure 4:**
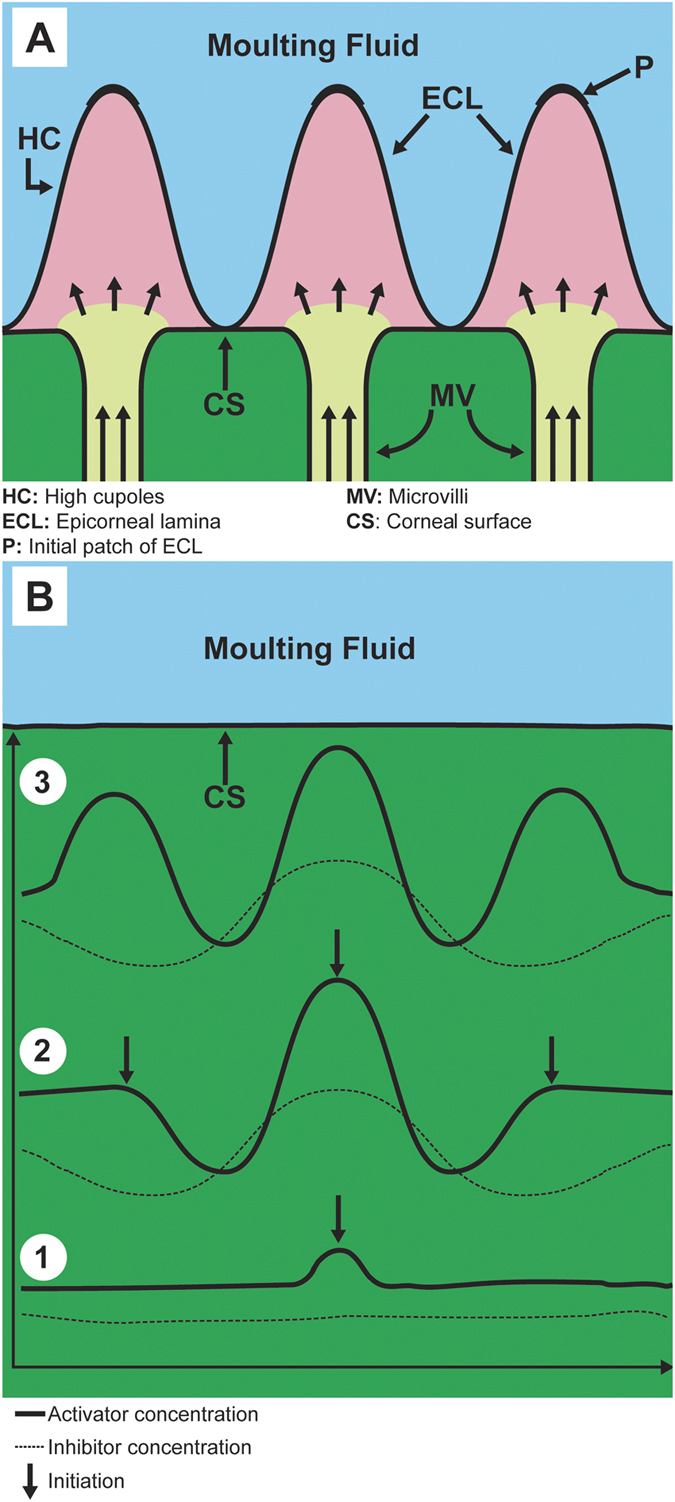
Comparison between two proposed theories for nano-nipple formation. (**A**) Schematic diagram of nipple growth by microvilli secretion into preformed epicorneal lamina (modified after Gemne (5)), and (**B**) diagram showing a sequence (steps 1–3) of fluctuation in activator and inhibitor concentrations that correspond to the profiles of nano-nipples (modified after Kondo and Miura (21)).

While this study is not conducive to provide definite answers as to specifics of the growth of the nipple structure, it serves to provide significant new structural insight to test if the final observed structure is consistent with the two currently considered growth models: the microvilli model proposed by Gemne^5^ and the Turing model proposed by Blagodatski *et al*.^8^. Again, it should be emphasized that one of the main differences between the two models is the flow of lens-forming material during the formation of the nipples as shown schematically in Fig. 4. In the case of the microvilli model the flow of material is from the inside of the lens cone, filling pre-existing cupole cavities above the lens surface (Fig. 4A). The 75–90 nm diameter microvilli are clearly visible in the micrographs presented in the study by Gemne^5^. In many respects this growth mode is analogous to a mold filling process. On the other hand, in the Turing model (Fig. 4B) proposed by Blagodatski *et al*.^8^ the flow direction of material precursors (morphogens) is less defined and was described to occur in the colloidal or liquid crystal like environment in the developing eye^8^.

The challenge for the microvilli model is that it must explain the formation of domain/grain boundaries with very specific configurations – dominated by rows of 5–7 defects – in the final nipple structures. This model requires that the root-cause of the specific nipple arrangements is the initial arrangement of microvilli before actual nipples are formed inside the cupole cavities. Space filling arguments used in other 2D hexagonal structures could be helpful. Given the fact that each ommatidium has some curvature, the problem of lattice compression or stretching arises in the nipple arrays. This effect is known as topological or geometric frustration (e.g. refs 27 and 28). Many different systems of this type have been studied in great detail, and one commonly observed topological defect that can reduce the associated lattice strain is the coordination defect either individually as 5-fold or 7-fold disclinations or in combinations such as the 5–7 defects, which have a dislocation character^27^,^33^,^55^. Examples of such defects were found in many structures including colloidal systems, charged particles, convection patterns, carbon fullerenes, or anodized aluminum pores^47^. Various 5–7 based line defects were even found on flat 2D hexagonal crystals as a stress relieving mechanism^31^,^43^,^45^,^56^,^57^. Therefore hexagonally arranged microvilli containing coordination defects and grain boundaries together with some elastic lattice bending could be easily envisioned.

The Turing model proposed by Blagodatski *et al*.^8^, on the other hand, involves the reaction and diffusion of chemical species known as activator and inhibitor within a developing tissue^18^,^19^. Furthermore, the pattern formation depends strongly on their concentrations and diffusion coefficients. According to Kondo and Miura^21^, two conditions must be met to initiate pattern formation: i) the inhibitor must diffuse faster than the activator, and ii) there needs to be a local change in activator/inhibitor concentration (Fig. 4B1). Given that the first condition is met, a cascade of pattern formation could be triggered by a local fluctuation of activator/inhibitor concentration. As a result, the nipple “nucleation” could potentially be initiated in numerous locations, and the surface would ultimately be filled with multiple sets of ordered hexagonal arrays. What is required to test this model is Turing type modeling covering sufficiently large areas over the ommatidium. The work by Blagodatski *et al*.^8^ showed Turing patterns for a variety of possible diffusion/reaction conditions together with their insect eye counterparts. The results were fascinating in that the entire set of possible surface structures observed on insect eyes can be obtained by using varying sets of variables in the diffusion reaction model.

For the case of the highly ordered hexagonal structure an atomic force micrograph of Lepidoptera nipple structure was shown by Blagodatski *et al*. covering an area of about 11 × 11 nano-nipples^8^. This micrograph clearly shows the presence of two 5–7 coordination defect pairs very similar to the ones described in our current study. On the other hand, the diffusion reaction modeled pattern that corresponds to the highly ordered structure presented in the main part of the Blagodatski *et al*. study depicts a slightly smaller area of about 8 × 8 nipples (or roughly 1.6 × 1.6 μm, if scaled with the unit cell size of 0.025 nm, Table 1) showing mainly perfectly ordered hexagonal structure. However, on the right hand side of the pattern some deviation from lattice regularity in the form of lattice bending and slight displacement of two of the nipples from their regular lattice sites is observed. Careful analysis of the multitude of modeled structures presented in the supplemental material by Blagodatski *et al*.^8^ shows that some of the modeled structures do contain pairs of 5–7 coordination defects.

**Figure 5:**
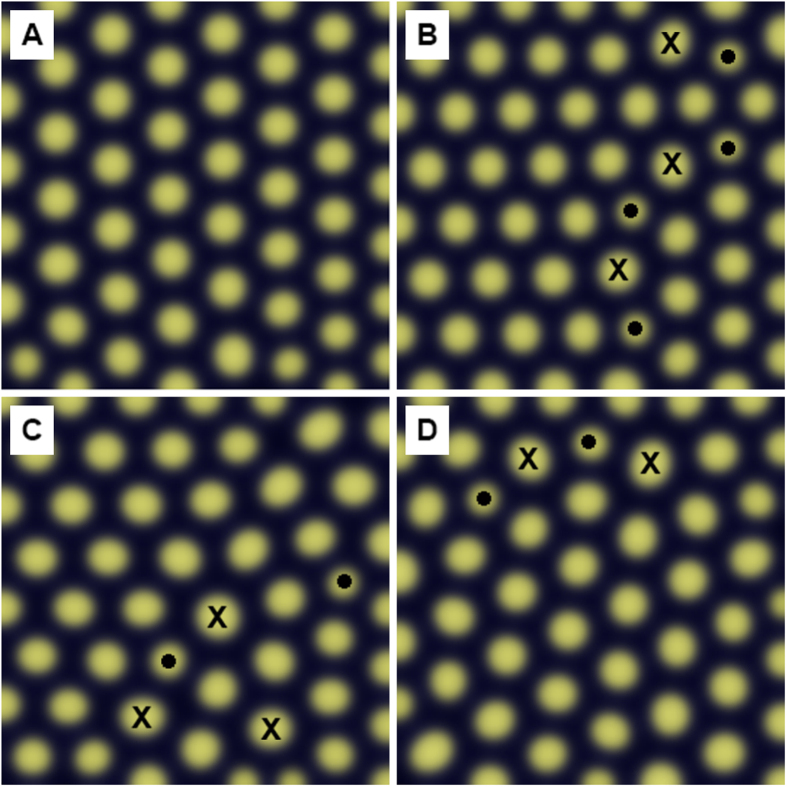
Patterns generated using the reaction-diffusion model using the following parameters taken from the work by Blagodatski *et al*. (8): d_u_ = 0.03, D_u_ = 0.04, a_u_ = 0.08, b_u_ = −0.06, c_u_ = 0.04, F_max_ = 0.2, d_v_ = 0.08, D_v_ = 0.6, a_v_ = 0.19, b_v_ = 0, c_v_ = −0.15, G_max_ = 0.5. Under the same conditions, the model generates random patterns with varying structural order (**A**–**D**).

In order to check that similar results as presented in the current study can indeed be obtained from the diffusion reaction model, we used the code presented in the work by Kondo and Miura^21^ and applied parameters for D_u_, D_v_, b_u_ and a_v_ used by Blagodatski *et al*.^8^ for the region in which they found highly ordered structures. Multiple calculations were performed and four random cases of final results are shown in Fig. 5. Each area corresponds to a size of about 7 × 7 lattice points and, given the lattice parameter of 0.205 μm (Table 1), an area of about 1.4 × 1.4 μm^2^, the average grain size observed in this study (Fig. 3A).

Figure 5A shows a pattern of structure with largely single crystal character. There is some minor lattice bending when looking at the rows of closest packed lattice points. Note however, that the lattice point at the bottom left corner is significantly displaced from its regular lattice position. Figure 5B–D show three examples of several final patterns that do indeed contain the essential features as shown in the scanning electron micrographs presented in the current study. These features are elastic lattice distortions to varying degree as well as 5–7 coordination defects aligned in rows, again as indicated in Fig. 5 by “•” and “×” Given that the average grain/domain size on the Mourning Cloak butterfly was 1.4 μm, which is smaller than 36% of the crystals (Fig. 3B), these patterns are compatible with the results from the SEM studies. Depending on which 1.4 × 1.4 μm^2^ area is randomly chosen on the SEM micrographs, regions which are nearly defect free or contain a few aligned coordination defects together with some elastic lattice bending as shown in Fig. 5 could be expected.

**Figure 6:**
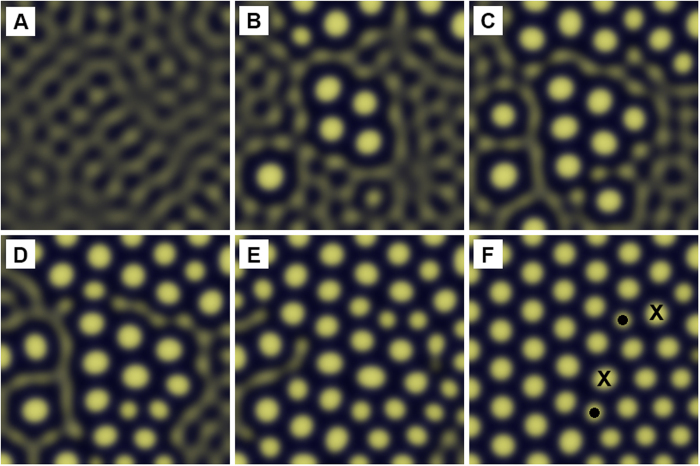
Snapshots of Turing pattern formation with increasing time (**A**–**F**). (**A**) Pattern formation begins with weakly defined structure, (**B**) nuclei begin to appear in several locations, (**C**–**E**) close-packed pattern begins to emerge and builds on existing nuclei, and (**F**) is the final pattern containing a row of closely spaced 5–7 defects.

With respect to the development of the Turing pattern as a function of time, Fig. 6 shows a sequence of snapshots in the pattern formation obtained for the same conditions as used in Fig. 5. The pattern is first nucleated in several areas as individual crystals (Fig. 6B). With increasing time the nuclei grow and join together to form a final pattern containing 2 pairs of 5–7 defects aligned in a row, which forms a high angle grain boundary between two crystals in different orientations. Two other examples of similar pattern development showing final structures with two 5–7 defect rows and a single crystal with a 5-fold coordination defect, respectively, are shown in Figs S4 and S5 in the supporting material.

Closer inspection of both Figs 5 and 6 also shows one other important feature in the final structures. The 5-fold coordinated motifs are usually slightly smaller than the motifs in regular 6-fold coordinated sites. On the other hand, the 7-fold coordinated motifs are usually slightly larger. This is also observed in the scanning electron micrographs of the nipple structures for the Mourning Cloak butterfly as discussed in our earlier analysis of the nanoscale structure for this butterfly^22^.

In summary, the mesostructure analysis of the highly ordered hexagonal corneal nipple arrangement on the butterfly *Nymphalis antiopa* presented here is consistent as an end result with both the earlier microvilli extrusion theory^5^ and the very recent Turing model theory of nipple coating development^8^, the latter not requiring the existence of microvilli. Perhaps both theories can be reconciled when the anlage formation in the very early stages of nipple structure formation is considered. The presence of microvilli cannot be ignored; however a one-to-one correspondence between microvilli positions and initial patch locations or final nipple positions (Fig. 4) is not always obvious in the electron micrographs presented by Gemne^5^. Perhaps the very existence of microvilli could enable Turing type pattern formation by providing pathways for control of activation and/or inhibitor concentration control during the eye development.

While the results of this study are of significant importance in the analysis and understanding of the intriguing nanostructure in nipple arrays of butterfly eyes, the findings are also equally important in the broader context of symmetry breaking 5- and 7-fold defects and their distribution in numerous other and seemingly unrelated structures referenced earlier^24^,^25^,^26^,^27^,^28^,^29^,^30^,^31^,^32^,^33^,^34^,^35^,^36^,^37^,^38^,^39^,^40^,^41^,^42^,^43^,^44^,^45^,^46^,^47^,^48^. The type of 5- and 7-coordination defect and combinations thereof to form higher order defects have been observed in 2D structure systems such as magnetic bubble arrays, block copolymers, convection patterns, charge distributions, graphene, etc. However, all of the previous studies have looked at relatively small sections of specific structure systems. The current study is likely the first report ever in which the structure of such a system has been described in its entirety including and beyond the natural boundaries surrounding the system, in this case individual ommatidia and their neighbours separated by relatively poorly structured ommatidia boundaries.

## Methods

### Scanning Electron Microscopy (SEM)

Images presented in this study were taken using a Hitachi S4500 field emission electron microscope at an accelerating voltage of 1.5 kV and a working distance of 5 mm by capturing secondary electrons. Butterfly eye samples were separated from the butterflies supplied by Thorne’s Insect Shoppe located in London, Ontario, Canada and then affixed by carbon paint on SEM sample stubs. Carbon coatings were applied to butterfly eye samples by cathodic arc deposition to mitigate charging effects.

### Orientation Mapping

Orientation mapping was done using Image J with Orientation Mapping plug-in^49^. The area occupied by each color was determined using “Threshold_Color” plug-in.

### Crystal Size Measurements

Grain boundaries were first defined by tracing aligned 5–7 defect rows (Fig. 2C). Areas of individual crystals were then determined using Image J, which calculates the area occupied by user-defined shapes. Based on the area, the crystal sizes (d) were approximated assuming circular grain shapes as follows:
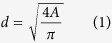


where *A* is the crystal area and *d* is the crystal size. The results were presented using a histogram (Fig. 3) in Microsoft Excel to show the distribution of crystal sizes. Because the histogram is asymmetric and follows the trend of a log-normal distribution, a best-fit was found according to the theoretical log-normal probability distribution function:
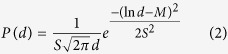


where *d* represents crystal sizes at which the probability is evaluated and ranges from 0 to 5 in 0.1 μm increments, *M* is the mean of the natural logarithm of the measured data (*d*), and *S* is the standard deviation of the natural logarithm of the same data set. As a result, the best-fit was found when *M* = 0.229, *S* = 0.452, and *C* = 55.59, and it is represented by the red curve in Fig. 3A.

## Additional Information

**How to cite this article**: Lee, K. C. *et al*. Mesostructure of Ordered Corneal Nano-nipple Arrays: The Role of 5–7 Coordination Defects. *Sci. Rep.* **6**, 28342; doi: 10.1038/srep28342 (2016).

## Supplementary Material

Supplementary Information

## Figures and Tables

**Table 1:** Summary of feature dimensions in the Mourning Cloak butterfly eye.

Major axis of eye (μm)	2.2 × 10^3^	Lattice parameter (μm)	0.205
Minor axis of eye (μm)	1.6 × 10^2^	Average nipple diameter (μm)	0.17
Surface area of an eye (μm^2^)	5.06 × 10^6^	Average nipple area (μm^2^)	2.27 × 10^−2^
Number of facets	1.04 × 10^4^	Nipple density (/μm^2^)	27.5
Average facet area (μm^2^)	4.9 × 10^2^	Estimated total number of nipples	1.4 × 10^8^
Average grain size (μm)	1.4	Average number of nipples per facet	1.34 × 10^4^

**Table 2:** Area of colored regions in the orientation map.

Orientation Range	Total Area (**μm**^**2**^)	Percentage (**%**)
0°–10°	73	16
10°–20°	79	18
20°–30°	79	18
30°–40°	65	14
40°–50°	64	14
50°–60°	90	20
Total	450	100
