# Supplementary material for: Mesostructure of Ordered Corneal Nano-nipple Arrays: The Role of 5–7 Coordination Defects
Source: Sci Rep. 2016 Jun 22;6:28342. doi: 10.1038/srep28342 (PMC4916435; doi:10.1038/srep28342)
Supplement: Supplementary Information [file srep28342-s1.pdf]

Supplementary Information

Title: Mesostructure of Ordered Corneal Nano-nipple Arrays: The Role of 5-7 Coordination Defects

Ken C. Lee, Qi Yu, Uwe Erb*
Department of Materials Science and Engineering, University of Toronto, Canada
184 College St. Toronto, Ontario, Canada M5S3E4

Supporting Material
S1 Text

Figure S1A represents a defect-free region in one of the crystals/domains showing the hexagonal unit cell in the same orientation at two different locations. The lattice parameter and unit cell area are 0.205 µm and 3.64 x 10^{-2} μm^2, respectively (Table 1). With an average nipple diameter of 0.17 µm the average nipple density is 27.5 per μm^2 this translates to about 1.4 x 10^8 nano-nipples for the entire eye surface.

Figure S1B shows another area on the same ommatidium at the same magnification as Figure S1A. However, in this case the area contains defects. It is clearly seen that in this region the unit cells on the left and right are in different orientations. Figure S1C shows the same region as Figure S1B but now with the defects marked with "•" and "×," indicating the positions of the 5-7 defect pairs which are responsible for the lattice rotation seen in Figure S1B.

The 5-7 defects are aligned in a row from the top to the bottom as shown in Figure S1D, where the two crystals/domains are divided by the defects in a similar way as crystals are divided by grain boundaries in engineering materials. Such observations led us to use grain boundary models to describe aligned 5-7 defects (1). In the case of Figure S1D, the defect row can be described using the dislocation model based on the defects' similarity to a dislocation array (1, 2). The model predicts an inverse relationship between defect spacing and the degree of misorientation based on the equation $\theta = \sin^{-1}\left(\frac{b}{d}\right)$, where θ is the degree of misorientation, b is the lattice parameter, and d is the spacing between 5-7 defects. This specific boundary is a low-angle boundary as it causes a relatively small misorientation of approximately 15°, which is in good agreement with the approximation of the dislocation model**.** For other 5-7 rows, other grain boundary models also apply depending on 5-7 defect pair spacing and arrangement (1). These include the structural units model for grain misorientations larger than 15° (1) as well as the coincidence site lattice model (3).

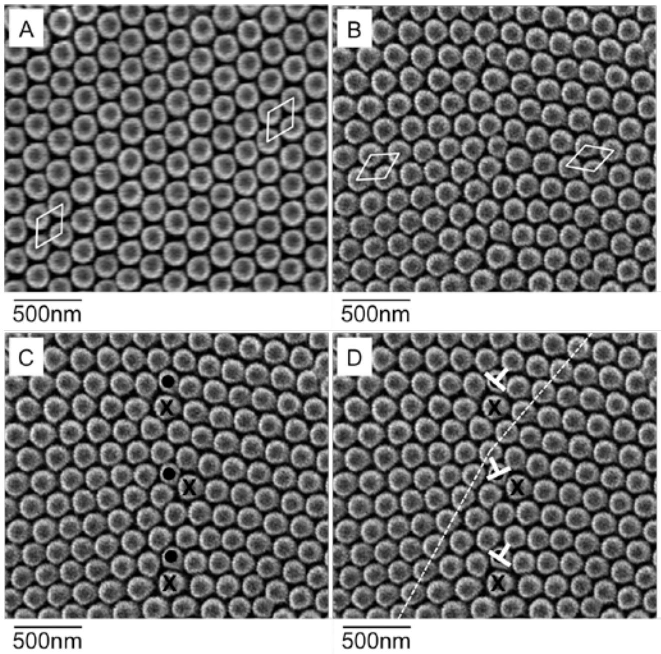

Figure S1

Fig. S1. Crystal and defect structure of the Mourning Cloak butterfly eye nipple array. (A) A defect-free nipple array with unit cells indicating the same lattice orientation in different locations, while (B) shows an area containing 5-7 defects with unit cells indicating local variations in lattice orientations. The 5-7 defects are labeled in (C) and divides the two areas in different orientations. Based on the dislocation model, this row of defects is similar to a dislocation boundary in crystallography (D).

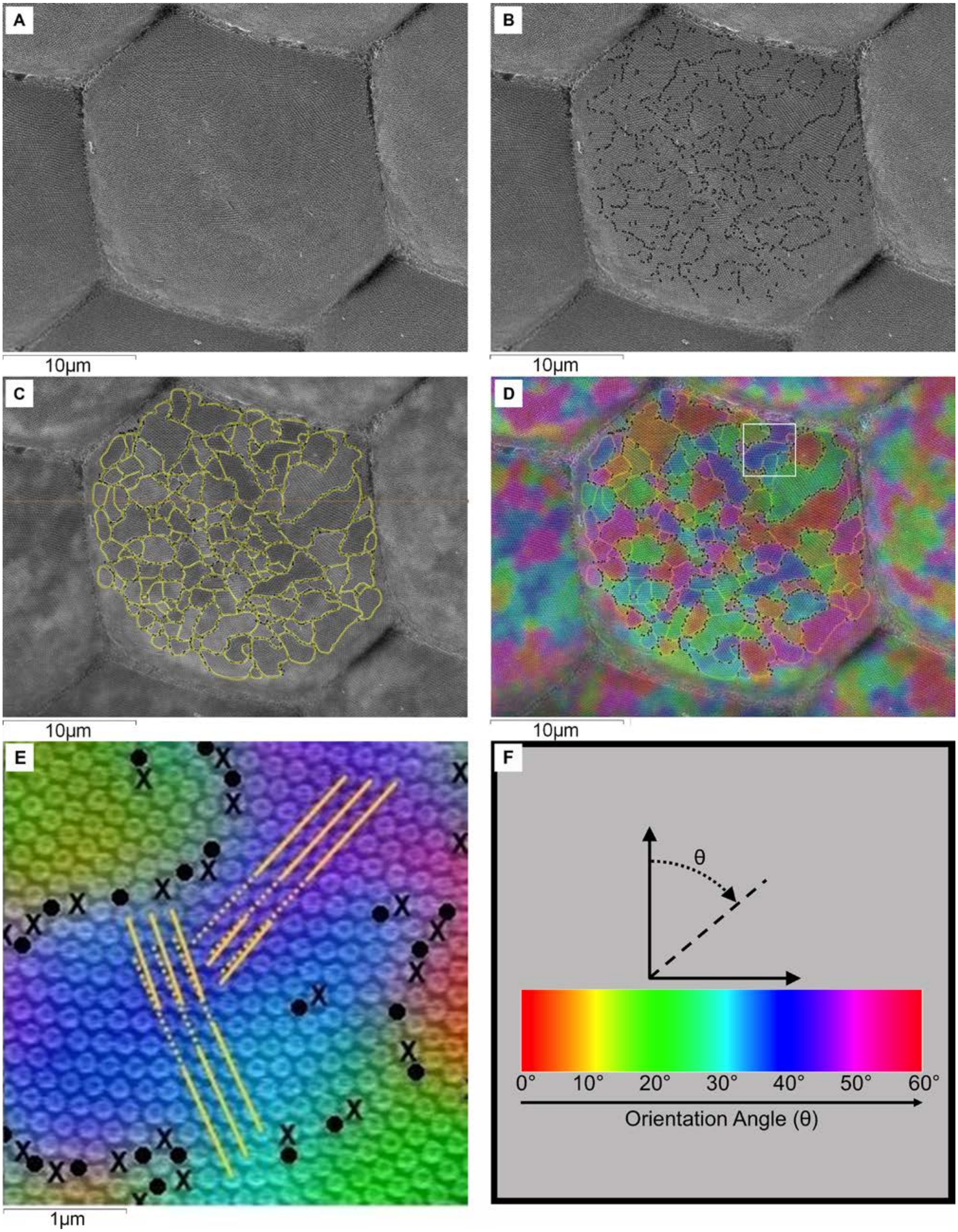
A
B
10μm
10μm
C
D
10μm
10μm
E
F
θ
0°
10°
20°
30°
40°
50°
60°
Orientation Angle (θ)
1μm

Figure S2

Fig. S2. Ommatidium showing defined nipple crystals. (A-D) show the same ommatidium, and the nipple crystals become visible after all 5-7 defects are labeled (B). The crystal definition is further improved by tracing 5-7 defect rows with solid green lines (C). (D) shows the orientation map of the ommatidium, which is to be interpreted based on the color wheel in (F). (E) is a close-up of the area outlined in white in (D), clearly showing the effectiveness of orientation mapping to capture elastic lattice bending.

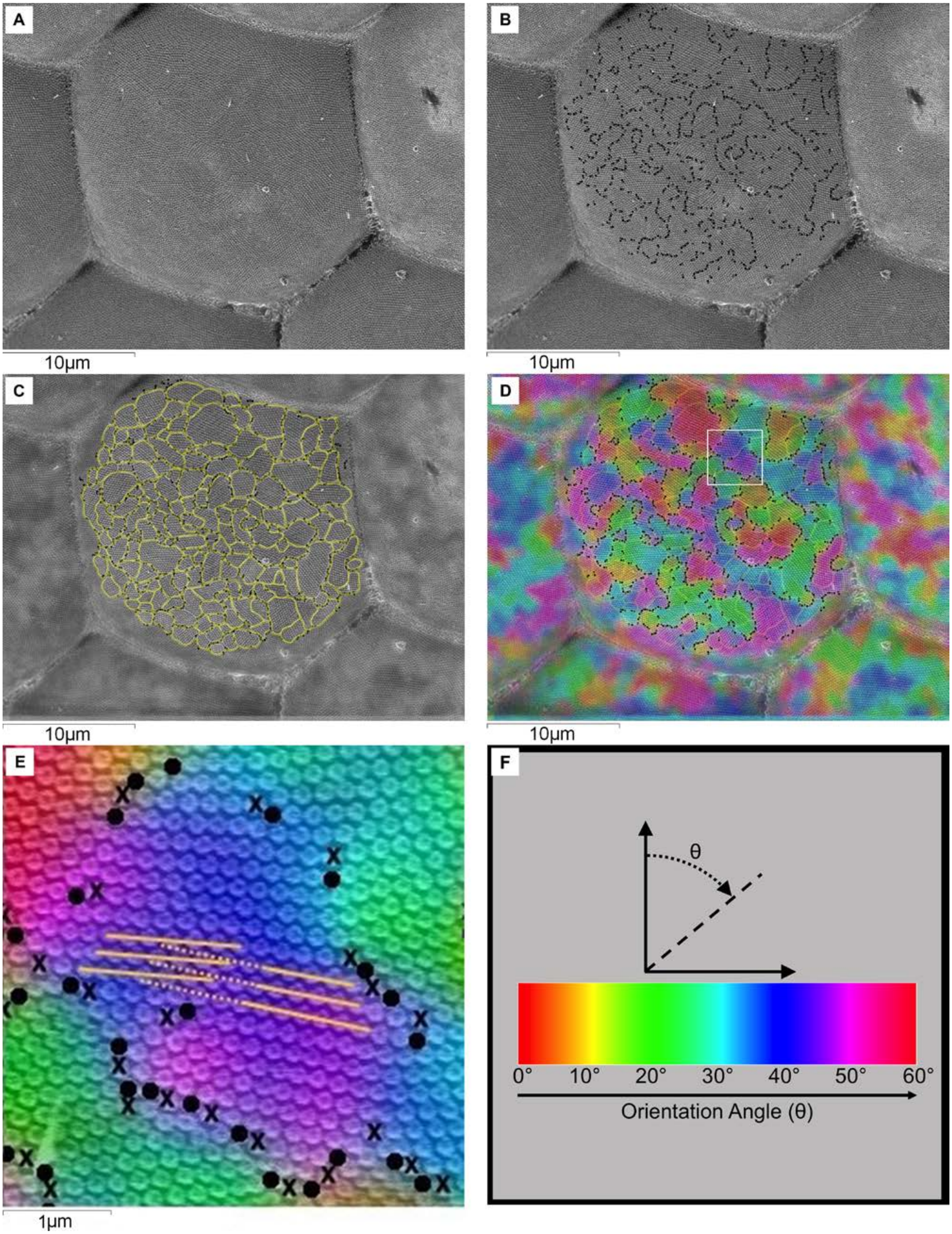
A
B
10μm
10μm
C
D
10μm
10μm
E
F
θ
0°
10°
20°
30°
40°
50°
60°
Orientation Angle (θ)
1μm

Figure S3

Fig. S3. Ommatidium showing defined nipple crystals. (A-D) show the same ommatidium, and the nipple crystals become visible after all 5-7 defects are labeled (B). The crystal definition is further improved by tracing 5-7 defect rows with solid green lines (C). (D) shows the orientation map of the ommatidium, which is to be interpreted based on the color wheel in (F). (E) is a close-up of the area outlined in white in (D), clearly showing the effectiveness of orientation mapping to capture elastic lattice bending.

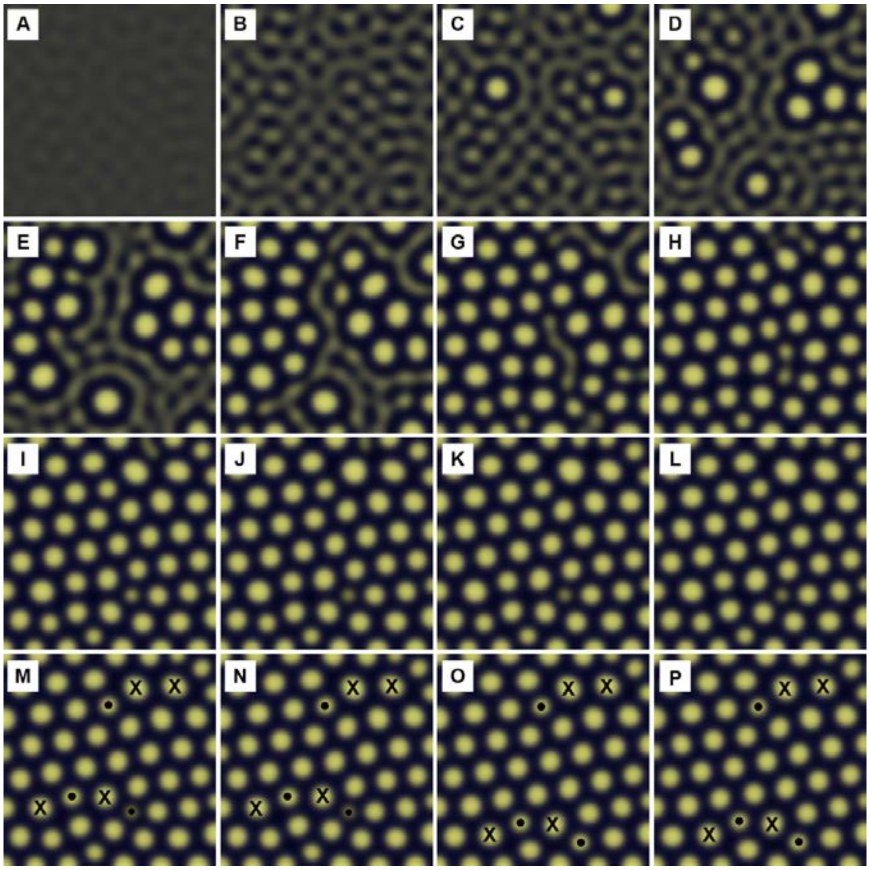

Figure S4

Fig. S4. Snapshots in the sequence of Turing pattern formation using the program provided by Kondo and Miura (4) using the same parameters as in Figure 6, derived from the work by Blagodatski et al. (5). Images were captured at different time steps in the formation process. (A, B) are the initial stages of the process where the pattern emerged slowly, therefore time increments of t~100,000 each were given to develop. (C-N) show much more variation in the patterns. Each step is captured at smaller time increments (t~25,000) to show pattern progression in more detail. Once stability has been reached (O, P), the pattern showed little change and was captured at t~100,000 increments. However, it is important to point out that pattern variation could still occur in the late stages of formation between (N) and (O), where the 5-7 defect row near the bottom migrated towards the edge of the figure.

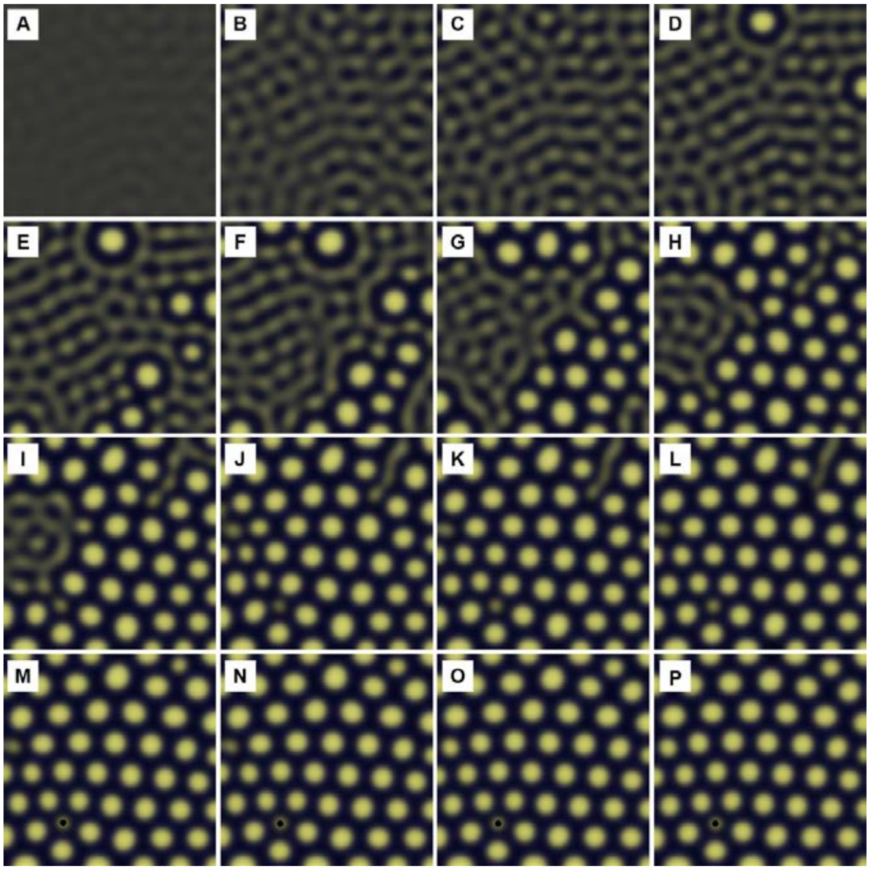

Figure S5

Fig. S5. Snapshots in the sequence of Turing pattern formation using the program provided by Kondo and Miura (4) using the same parameters as in Figure 6, derived from the work by Blagodatski et al. (5). Images were captured at different time steps in the formation process. (A, B) are the initial stages of the process where the pattern emerged slowly, therefore increments of t~100,000 each to develop. (C-N) show much more variation in the patterns. To show pattern progression in more detail, each step is captured at smaller time increments (t~25,000). Once stability has been reached (O, P), the pattern showed little change and was captured at t~100,000 increments. Compared with Fig. S4, the non-spherical portion of the pattern remained for much longer; this shows the random nature of the Turing model even while using the same parameters.

References

1. Lee, K. C. & Erb, U. Remarkable crystal and defect structures in butterfly eye nano-nipple arrays. *Arthropod Struct. Dev.* **44**, 587-594 (2015).
2. Li, J. C. High-angle tilt boundary – A dislocation core model. *J. App. Phys.* **32**, 525-541 (1961).
3. Lee, K. C. & Erb, U. Grain boundaries and coincidence site lattices in the corneal nanonipple structure of the Mourning Cloak butterfly. *Beilstein J. Nanotechnol.* **4**, 292-299 (2013).
4. Kondo, S. & Miura, T. Reaction-diffusion model as a framework for understanding biological pattern formation. *Science* **329**, 1616-1620 (2010).
5. Blagodatski, A., Sergeev, A., Kryuchkov, M., Lopatina, Y. & Katanaev, V. L. Diverse set of Turing nanopatterns coat corneae across insect lineages. *Proc. Natl. Acad. Sci. U. S. A.* **112**, 10750-10755 (2015).
